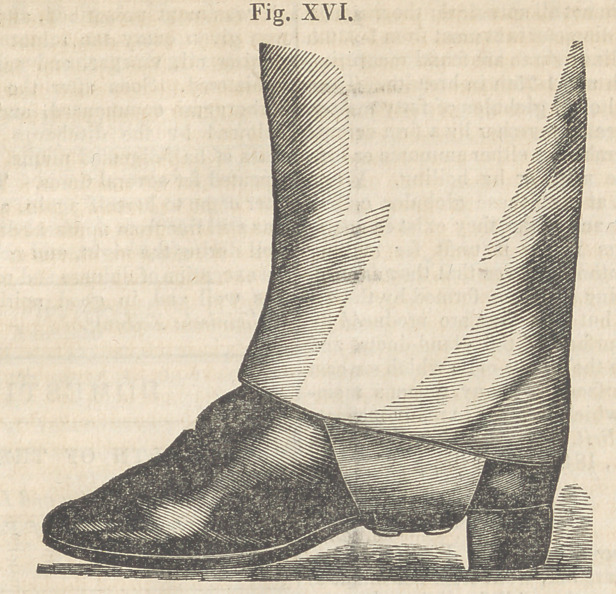# Second Report of Cases of Deformed Feet, Treated without the Division of Tendons, with a Description of the Apparatus Employed

**Published:** 1841-03-13

**Authors:** Heber Chase

**Affiliations:** Philadelphia


*Second Report of Cases of Deformed Feet, treated without the Division of Tendons, with a description of the Apparatus employed.* By Heber Chase, M. D., of Philadelphia.

In deformities of the feet, whether there exist an inversion or eversion, the same principles will apply to their treatment. In these cases, whether the foot has advanced to the first, second, or third degree of *varus*, as described by authors, the first step towards a restoration, consists in bringing the distorted foot into the same axis with the leg. This we have accomplished by means of an instrument represented in fig. 1. It consists of two parts, a brass splint, (*a*,) and a steel plate, (*b*,) connected by means of a malleable iron neck, (*f*,) which can be bent, by considerable force, but will not yield to the power necessary to act upon the foot. The utility of this arrangement will be readily understood by the operator, because, in order to act to the greatest mechanical advantage upon the foot, the plate is required to be placed at different angles with the splint in different stages of the progress of restoration. The steel plate should be one inch in width for an adult, two lines in thickness, and extend to a distance equal to the interval between the ankle-joint and the ends of the toes.

In cases of inversion of the foot, the brass splint is applied to the outside of the leg. It should embrace one-third of the circumference of the limb, and should extend from just below the knee to the upper part of the external malleolus. It is secured to the limb by the straps, (*d, d.*)

Fig. I.

By means of this apparatus, the foot is brought outward towards the steel plate as far as possible, without occasioning much pain, and is then confined by the strap, (*e*,) which is thrown around the foot, and passed through the fenestræ, (*c*, *c*.)

In the progress of the restoration of the foot, the strap surrounding it requires to be drawn more firmly from time to time, as will be mentioned in the report of cases.

In *eversion* of the foot, the brass splint is to be adjusted to the *inner side of the leg*, when the same principles will apply as in *inversion.*

The use of this instrument must be continued until the foot is brought into the same axis with the leg, and until the disposition to a return of the deformity has ceased.

The second indication to be fulfilled is to effect the proper flexion of the foot. This we have accomplished by means of the instrument represented in fig. II.

Fig. II.

It consists of a plate of brass, (*a*,) moulded to fit accurately to the back and sides of the leg, and extending from immediately below the knee to just above the malleolus. A second piece (*b*) formed to act as a sandal or shoe, equal in length, and a little wider than the foot. These are attached by a hinge, (*c*, *k*,) so as to admit of flexion and extension. The leg is secured in the brass splint, by straps, (*d*, *d*.) The foot is secured to the shoe by a strap, (*e*,) which is thrown around the instep, then passes through a fenestra behind the heel, and the extremities being reverted, are returned over the instep, where they are secured by a buckle. There is also a strap, (*g*,) intended to pass around the foot near the toes, in order to draw it outward, when flexion is being made. The fenestræ at the right and left of *b*, are for the passage of straps, when the instrument is employed in cases of eversion of the foot. *h*, *h*, are two straps for approximating the extremities of the instrument. *l*, a knob for securing the straps.

By examining the instrument itself, it will be seen, that the appendage marked *k*, does not clasp the shoe firmly, but stands out from it to the distance of half an inch on each side. This appendage passes beneath the shoe, and is attached by its centre, at a spot just anterior to the fenestra, (*f*,) by a universal joint with a limited motion. By means of this arrangement, when it becomes desirable to produce some degree of abduction of the foot in cases of inversion, this object may be accomplished by drawing the strap *h i*, more firmly than its fellow.

The leg is to be placed in the brass splint *a*, the foot in the shoe *b*, the leg is secured by the straps *d d*, the heel kept down by the strap *e*, and if desirable, the loop of the strap *g*, thrown around the foot in cases of varus, to produce partial abduction.

During the progress of restoration, the straps *h i*, and *h*, are to be drawn, from day to day, more tense, as the foot yields to the action of the instrument.

In deformities of the feet varying from those above mentioned, the instrument employed, will be described in connection with the cases.

CASE I.—*David Canning Smith aged* 17 *years —Congenital inversion of the right foot, of the worst variety—supposed to have arisen from a blow during Utero-Gestation—Brought to a direct linewith the leg by abduction, in two weeks—Flexed to the position seen in Fig.* IV, *in twenty days, by mechanical means alone.*

The young gentleman who is the subject of this case, although of the age above mentioned, possesses a frame of unusual size and developement. His father is a remarkably powerful, robust, and healthy man, while his mother, on the conrtary, is of small stature. They have had nine children, all of whom, with the exception of David, are well formed in their limbs.

Five weeks previous to her confinement the mother accidentally received a blow on the abdomen from one of her daughters, and the child was not felt to move for several days as it had done before.

On the birth of the infant his right foot was inverted to a considerable degree, and the skin, as stated by the mother, exhibited a dark livid appearance over the external malleolus.*

The parents at this time resided in the vicinity of Dublin, where the child was taken at an early age, and attempts were made to restore the limb, but every thing proving ineffectual, the case was abandoned, and the foot soon assumed the position seen in Fig. III.

Fig. III.

Fig. IV.

At the usual age he began to walk—was always active; and at the time I first saw him, which was in the early part of the month of September, 1840, he was engaged as a salesman in a house of business in this city.

His foot was so far inverted that the toes pointed directly toward that of the opposite side, and so rotated that he walked on the outer side of the foot, which had formed for itself a perfect cushion upon which it rested, turning backward, while the heel was elevated a little more than an inch, a circumstance which necessarily follows the inversion and rotation of the foot to the extent spoken of by writers, under the head of *varus* of the third degree. This elevation of the heel, however, becomes restored whenever the foot is brought to the same axis with the leg, followed by flexion. He had no direct command over the

---

*It is a well known fact that many ingenious theories, and speculations have been advanced to explain the cause of deformities of the feet.

Independent of the case cited above, I have lately treated a child of eight months old, in which an inversion of both feet of the worst variety existed, attributed by the mother to the following cause:—

When in the fifth month of her pregnancy, while walking upon the flat of the house, she accidentally slipped, and fell with her feet doubled under her, which gave her considerable pain for several hours.

The idea that her child might have deformed feet immdiately seized upon her mind; and her mental anxiety was in a few days heightened by the circumstance of a carpenter's coming into the neigbourhood who had an inverted club-foot, and who was employed in finishing a house on the opposite side of the street, just in view of her parlor windows; and to use her own expression, "I never could look into the street but I saw the man with the club-foot."

A few weeks ago I attended a young woman in her first confinement, whose infant was born with double calcanean club-feet.

The dorsa of the feet were thrown backward upon the lower part of the leg—the left, making a strong impression in the sub-cutaneous fat, and cellular tissue along the tibia. The soles of these feet were convex transversely, and to some extent, antero-posteriorly; in short, they had the appearance of having been moulded into the concave surface of a sphere.

The child is healthy, and in every other respect well proportioned, and no external cause can be assigned for this mal-formation.

foot, not even as much as to enable him to move his toes.

As is usual in these cases of deformity, though, perhaps, not to so great a degree in all, the foot was two inches shorter than its fellow, but well proportioned. Its circumference, embracing the heel and the instep, was one inch less—at the hollow, three-fourths—while just posterior to the toes, it was of the same measurement as the foot of the opposite side.

The leg was half an inch shorter than its fellow, wanting development at the calf, and has always been of a lower temperature than the other extremities, so that during the cold weather in winter he has for two years past found it difficult to keep the limb warm.

There was no defect in the knee, except that it was a little smaller, and no change in the position; but its patella had a greater mobility than that of the other.

On the 22d of September, 1840, I began to abduct the foot by means of the instrument described (Fig. I.) and employed as there directed, drawing the strap (*e*) more and more tense from day to day; and in two weeks brought it into the same axis wtih the leg. This instrument was continued three weeks, until, by its constant action, the toes were made to evert, as is observed in a well directed foot when planted on the ground; which action was continued until all disposition of the foot to return to its original position had ceased.

Flexion was now begun, and in twenty days from the time of the application of the instrument (see Fig. II.) the foot was brought to assume the position seen in Fig IV.

*January*, 1841.—This patient is now able to walk with comparative ease. The temperature of his foot has become natural, and has improved during treatment in proportion as the foot was restored; and the motion at the instep is also daily increasing. The rigidity which formerly existed in the fascia plantaris and adductor tendons, has subsided.

In consequence of the regularly graduated power which was employed in the restoration of the foot, but little pain was experienced during the treatment; and this was felt only on the right side of the foot in abduction, and at the instep in flexion; not any whatever in the tendo Achilles, nor has this tendon been rendered unusually tense by mechanical efforts upon the foot.

*March 4th.*—The patient can abduct, adduct, or extend this foot as readily and freely as the other; he can also flex it, and elevate his toes four inches. This motion of the foot allows tolerably free action in walking, but the flexion will daily improve as the foot acquires its full power.

The arch of the foot is higher than its fellow, but not so regularly formed as if no deformity had ever existed—as will be seen by reference to Fig. IV.

He can walk the distance of a mile with little or no fatigue, nor does he limp or hesitate in his movements.

He has been treated throughout in presence of my class last winter; and several medical gentlemen have examined the foot since its restoration.

Case II. *Congenital Calcanean Club-foot of the left side; restoration of the foot to a natural position by mechanical means, in twenty days.*

On the 13th of July, 1840, J. B. H., Esq., of this city, requested me to see his little son, a healthy, robust child, four weeks old, and whom I found to have a deformity of the left foot—(calcanean club-foot of the worst variety.) The deformity is congenital—no cause can be assigned for it. Mrs. H. is the mother of several children, all of whom are perfect in their limbs, nor can there be traced a deformity either in the paternal or maternal branches of the family, both of which are numerous.

The dorsum of the foot was drawn upward, in such a manner as to rest firmly upon the lower part of the front of the leg, whilst an obliquity caused the small toe to rest on a line with the inner side of the leg. See Fig. V.

Fig. V.

From the tender age of the child, it was not to be expected that much rigidity of the misplaced parts could have taken place as yet; therefore, the foot could be brought nearly to its true position, by moderate force applied to it by the hand, whilst the leg and ankle were supported; but returned immediately to its distorted position when these efforts ceased.

There was some want of development in the foot and leg generally, when compared with its fellow; and at the lower part of the leg, where the dorsum of the foot rested upon it, the subcutaneous fat and cellular tissue were to a considerable degree wanting, and the leg, when the foot was elevated, presented a perfect cast of the dorsum of the foot.

For the relief of this deformity, I applied to the outer side of the leg, for the purpose of bringing the foot not only downward, but inward, an instrument extending from the knee to the bottom of the foot similar to that represented in Fig. I, with the plate *c*, bent at a right angle with the splint at *f*, and secured by the straps *b b*. The foot was then brought down to half the distance required for restoration, and secured by a roller passing round it and through the fenestræ.

For a few days I saw this patient daily, afterwards, less frequently. At each visit I adjusted the instrument when necessary, bringing the foot nearer to the required degree of extension and eversion until the second of August, when it was brought to a correct position as seen in Fig. VI., and remained so when the instrument was removed.

*Aug.* 15*th.* The patient has apparently suffered very little from the dressings. Not even an abrasion of the skin has followed the use of the instrument, and the child enjoys all the proper motions of the foot with perfect freedom.

Fig. VI.

*October* 16*th*, 1840. This little patient has been able, for several days past, to stand even upon his feet.

Case III.—*Congenital Inversion of the Right Foot, of the worst variety—*(*Varus of the third degree*)*—Treated with Complicated Machinery for several months, with little or no effect.—Restored in thirty-one days by a simple apparatus.*—Early in the autumn of 1839, my attention was called to Samuel M'Kee Chambers, ætat. 2, who had a complete inversion of the right foot. He was walking upon the outer edge of the foot, which had formed for itself a perfect cusion, upon which it rested—the sole turning backward whilst the toes pointed directly toward the opposite ankle. See Fig. VII. In addition to this inversion of the foot, there was a defect in the knee joint, permitting the leg to revolve upon the thigh, to the extent of one-fourth of a circle, and by the application of some force, the toes could be made to point directly backward. This seemed to be owing to a change in the cartilages of the joint, and the relaxation of the capsular and other ligaments. The leg itself was somewhat smaller than its fellow, but the thigh appeared of its natural dimension.

As soon as an apparatus could be prepared, I adjusted it to the foot of the child, and kept it in constant use, until May, 1840, when finding that very little progress had been made towards a permanent restoration of the foot, and that the patient was very unwilling to wear the machine, the instrument was laid aside. The leg, however, had commenced increasing in size—the knee had acquired some strength, and the limb was brought partially to its true position.

*July* 1*st*, 1840. Having now succeeded in the restoration of other cases of deformed feet re-

Fig. VII.

Fig. VIII.

quiring more difficult treatment, I again returned to my patient.

On the 3d of July, I applied an instrument similar to the first of those described in the introduction to this paper, (Fig. I,) and by the 10th, the foot was brought on a line with the leg. On the 12th, the apparatus for flexion, (Fig. II,) was adjusted, which brought the foot to the position as seen in Fig. VIII. in thirty-one days from the application of the first instrument.

Until the 15th of July, the foot was daily brought nearer to the desired position. Very little pain was experienced, no soreness was occasioned by the pressure, and the patient who is one of the most robust, obstinate, and restless of children, ran at large in the streets at will, during the whole treatment. An ordinary shoe was applied on the 12th of August.

This is the only instance of any deformity known to have occured in this family, either in the paternal or maternal branches, and no cause can be assigned for it by the parents.

Neither the tendons nor the fascia plantaris offer any resistance to the permanency of the foot as restored, nor do the tarsal or metatarsal bones exhibit that rigidity which so often limits motion, until a late period after the foot is brought to its natural shape.

The foot in walking assumes its proper position, and the patient does not limp, or hesitate at all in his movements. The arch of the instep is not defective and all the varied motions, even including abduction, are performed as perfectly as upon the opposite side.

*October 8th*, 1840. Two weeks ago I called to ascertain the situation of my patient's foot, and found him in the street, bare-footed, and was told he had been without his shoes for three weeks, No relapse from the original restoration had followed, and on the 15th inst. I exhibited this case to my class in a lecture on deformities.

Case IV. *Deformity of the Left Foot, not congenital, (Pes Equinus of the third degree, of six years' duration, combined with Partia Inversion of the front part of the foot,) restored in twenty-one days.*

The subject of this case, J. Arbuckle, ætat. eleven, was soon after birth observed, according to the statement of his parents, to have an unusual stiffness in the ankles, which, however, in his earlier years gave him no material inconvenience. He was remarkably healthy, very active, and walked at the usual age of childhood.

Occasionally, up to his fifth year, he complained of a pain in the hip for a short time. This difficulty never attracted the attention of his parents, particularly, until the foot was observed to turn gradually inward, and the heel to become elevated. Medical aid was then called. Bandages and splints were applied, but the deformity proving very obstinate, they were abandoned.

During the month of April of the present year, I saw the patient for the first time. There was, at this period, a partial inversion of the front part of the foot by rotation at the middle joint of the tarsus, while the distance from the toes to the heel was five and three-fourth inches; the direction of the foot being nearly perpendicular as seen in Fig. IX.

There was a want of development in the glutei muscles—the thigh, leg, and foot, were also smaller than those of the opposite side, and the whole foot was remarkably rigid. The tendo Achillis was very stiff, and the bones of the tarsus were prominent, as is seen in Fig. IX., and in short, the limb had undergone all the usual changes which take place, where it becomes necessary to sustain the weight of the body on the toes for a great length of time.

Fig. IX

Fig. X.

There was also a relaxation of the ligaments of the knee-joint, and while walking, the knee performed a peculiar rotatory motion outwards, which greatly retarded the patient's progress. These combined motions of the limb, together with the elevation of the foot, rendered it almost impossible for him to walk. He would frequently fall in the street, and after going a short distance, would suffer extreme pain in the foot and leg.

On the 11th of May, I applied to the outer side of the leg, the brass splint accurately moulded to the limb and the upper part of the external malleolus, extending from just below the knee to the last mentioned point.

After the application of this apparatus, the foot was drawn daily more and more toward the desired position, until, at the end of one week, it was brought into a direct line with the leg.

To fulfil the second indication—the flexion of the foot—the instrument represented in Fig. II. was applied. The apparatus was secured to the leg and foot, and bound firmly at the instep by means of the strap, (*f.*) The point of the sandal and the upper extremity of the splint at the knee, approximated daily by the aid of the two lateral straps connecting those points, until the 21st day after the second instrument was applied, when the foot was restored to its proper position.

After the first few days the patient was able to begin to walk, which accelerated the flexion.

The pain produced by these instruments throughout the whole operation was by no means worth regarding. The process of restoration was slow but constant, and the changes brought about so gradual, that not even an unpleasant sensation was experienced beyond an hour, at any one time during the treatment. Not the slightest inconvenience was felt in any of the tendons, not even in the tendo Achilis, duting the treatment, but the pain was confined to the outer side of the foot during the abduction, and to the instep during the flexion.

An abrasion of the skin took place and continued for a few days, being caused by frictions which were employed in aid of the treatment, but no such result was produced by the apparatus.

*September 28th.* There is still considerable rigidity in the instep. The motions of the foot are limited, and in walking, the rotatory motion of the knee is apparent. It is expected that support for the knee and continued exercise of the foot, will in time overcome these difficulties.

The condition of the foot, thirty days after the application of the first instrument, is shown in Fig. X.

This patient was seen, at different stages of the treatment, by Professor George McClellan, Drs. E. W. Leach, of Boston, Baldwin, of Georgia, and Drs. R. Coates, Brewer, and West, of this city.

Case V. *Greatly distorted Foot, from exposure, which commenced in early life, restored in fifty-one days, by mechanical means alone.*

In the spring of 1840, my attention was called to Julia Dunmore, who was standing upon her crutches and on one foot, resting herself. The patient is now fourteen and a half years old, healthy, and as active as could possibly be expected, with the deformity under which she labours. She was a remarkably healthy and unusually active child—walked readily when nine months old—but at the age of a year and a half, she entirely lost the use of her limbs from exposure in a damp cellar, was placed under medical treatment, and recovered the motion of her extremities except that of her right foot, so far as to be able to walk in six months, with the aid of one crutch, She retained this power for some time, when it was observed that the hip was enlaging, and the leg growing shorter. A second crutch was then obtained, and the patient began to place the foot to the ground. The ankle was still however weak, but she continued to rest upon this as well as on the opposite leg, in walking. The ankle continued giving way, until the foot was brought to the position seen in Fig. XI. and thus she remained when she came under my care.

The whole limb was at that period much emaciated, measuring only five inches in circumference at the ankle, six and a half at the knee, and eight inches at the largest circumference of the thigh. The hip, and in fact the right side of the body, partook of the general emaciation.

She could stand, but she could not walk without her crutches, and she was so feeble in her limbs, that when she fell she was compelled to crawl upon her knees, until she met with something by which she could raise herself up. The use of the limb produced great fatigue in it.

It would seem almost impossible that a greater deformity, or one more difficult of restoration, could exist, than is here shown. The foot was completely reversed. The patient rested the limb on the instep, which had been so long accustomed to pressure, that an enormous cushion (see Fig. XI, *a*,) had been formed to protect the foot from the ill-directed pressure.

In the treatment of this case, the same principles were to be applied as in the foregoing. The foot was first to be brought to the same axis with the leg, after which, flexion was to be made.

Accordingly, on Saturday, the 22d of May, 1840, I applied the brass splint to the outer side of the leg, as described in the preceding case. By the aid of the strap around the foot, I drew it daily nearer the line with the leg, until the tenth day, when it was made to assume the position seen in Fig. XII.

On Monday, June 15th, I commenced flexion, and succeeded at the end of fifty-one days, in bringing the foot to the position seen in Fig. XII.

The entire restitution of the natural position of this foot, was accomplished perhaps with less difficulty than would be presumed by observing it in its distorted state. This was owing to the relaxation of the ligaments, and the ease with which the bones moved upon each other.

Fig XI.

Fig. XII.

In the restoration of the foot, the pain experienced was comparatively little. In both rotation and flexion of the foot, this sensation was principally confined to the osseous structure.

With some effort this patient could bring her heel to the floor on the 10th of July, and on the 13th she began walking for the first time, and on the 30th the foot exhibited the appearance seen in Fig. XIII.

Fig. XIII.

That part of the foot on which it rested during the greatest degree of deformity, is now seen at *a*.

By reference to this figure it will be observed, that the leg is thrown slightly backward upon the foot, in consequence of a loss of proper action in the tarsus. This action the patient will again recover.

This patient has been seen by Drs. R. Coates, West, and Brewer; Drs. E. W. Leach, of Boston, and Baldwin, of Georgia.

Note.—*October 17th*, 1840. This patient has gained the use of her foot at the instep, and the leg is thrown forward to the proper position. She can walk several squares without much fatigue, and the general appearance of the foot is much improved from that seen in Fig. XIII. She was examined by my class on the 15th inst.

Case VI.—*Everted Deformed Foot; deformity commenced at two years of age, from paralysis; restoration in ninety days by mechanical means.*

During the month of February, 1840, Professors George and Samuel M'Clellan referred to my care Mr. J. B., aged twenty-five years, who was labouring under an everted deformed foot, as seen in Fig. XIV., the history of which, as given by the gentleman himself and his parents, is as follows:

He was a healthy, fat child, and walked readily at nine months old. At two years of age he was suddenly seized with paralysis of the lower extremities, and spasm of the muscles of the back of the neck: his head was drawn far backward, and remained immovable for several weeks. He could not walk or sit without support, and both legs became entirely useless. This state of things was followed by several months of severe illness, when the left limb gradually recovered. At three and a half years of age he could climb up by a chair. At eight years old he could walk a short distance by the aid of two crutches, and continued in this situation for eight or ten years. He then walked four or five years with a crutch and a cane, and afterwards with a cane only.

Neither of his ankles had entirely recovered from the paralysis when he began to bear his weight upon his feet; and as his general health improved, enabling him to take more exercise, his ankles, particularly the right one, gradually gave way, and assumed the appearance represented in the figure referred to.

The internal malleolus was very prominent, the bones of the instep rigid, the foot attenuated, and the leg and thigh much smaller than those of the opposite side. The left foot was also slightly everted. He had no control over his toes. In walking, the foot was thrown outward, resting upon the inner edge, and the internal malleolus came nearly to the ground. He suffered much pain in the ankle and leg in walking.

By considerable effort the foot could be brought inward nearly on a line with the leg, and in order to retain it permanently, a firm gaiter-boot was fitted to the foot and ankle. Two plates of steel were provided, three quarters of an inch wide, two lines in thickness, and attached at their upper extremities by means of a semicircular plate, designed to pass behind the leg near the kee. These were long enough to extend from the knee along each side of the leg to the bottom of the boot, beneath which they were bent and united by their extremities. Attached to the inner plate at the internal malleolus was a circular piece of steel plate three inches in diameter, about two lines in thickness at the circumference, and one-fourth of an inch at the centre. The whole being suitably padded was applied to the foot, and confined by straps passing round the leg.

Fig. XIV.

Fig. XV.

This boot and its appendages were worn for ninety days, when the foot assumed the position seen in Fig. XV.

The pressure at the internal malleolus by the circular steel plate was quite firm, and produced considerable pain in walking for the first few weeks. The rigidity of the ankle-bones limited the flexion of the foot for some time after it was brought to a line with the leg.

At this period, Sept 8th, the patient begins to enjoy the motions of the leg on the foot, and also to move his toes; the leg has increased one-fourth in size since the commencement of the treatment, and he is able to walk without any support, and without fatigue.

Drs. Bacon, Woodward, Smith, Morgan, Cogswell, and Ives of New England, are familiar with this case, and Professors Tully and Knight have examined the patient since his foot was restored—also the gentlemen who kindly referred him to my care.

Fig. XVI.

I have several patients under treatment for the cure of false anchylosis of the knee-joint, with different degrees of flexion. Three cases have been perfectly restored. Some of these deformities are of many years' standing. The results we propose to relate in a future number of this Journal.

## Figures and Tables

**Fig. I. f1:**
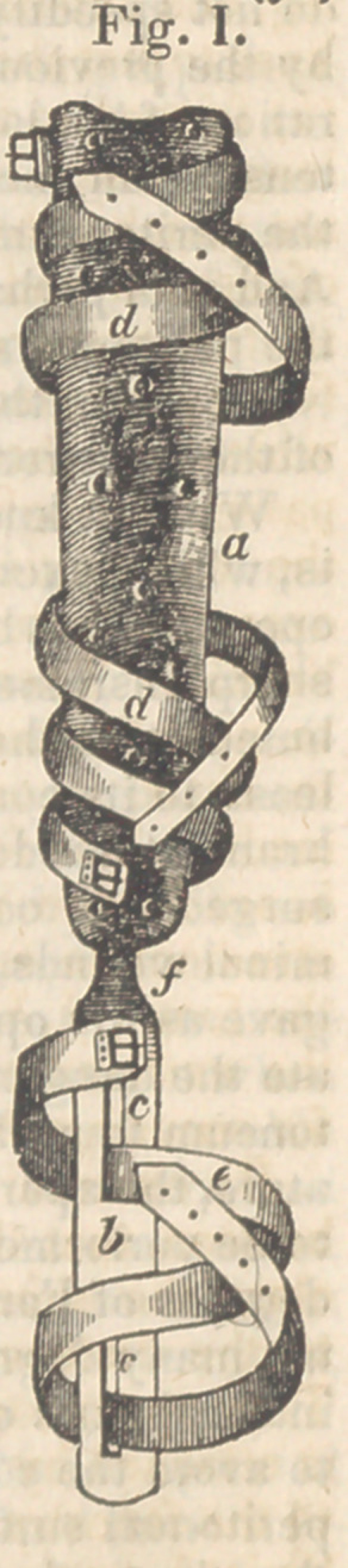


**Fig. II. f2:**
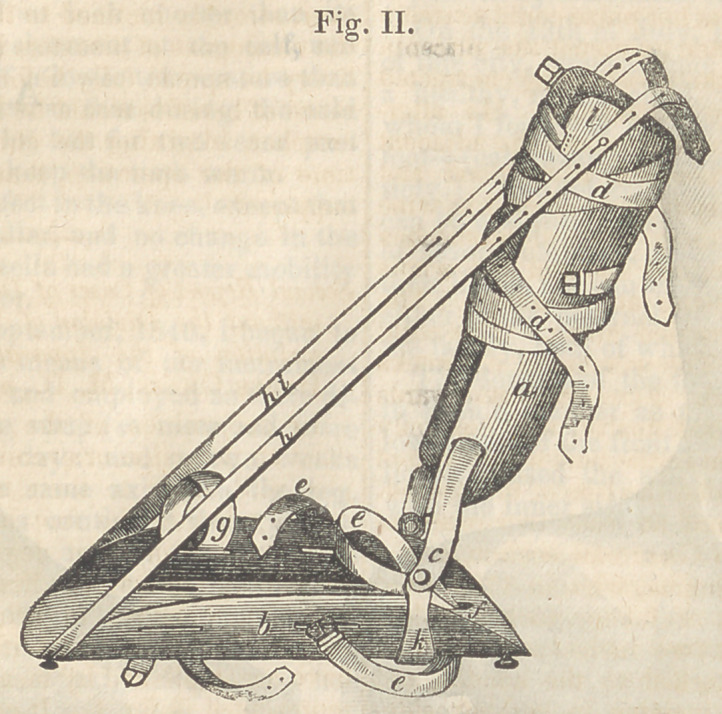


**Fig. III. f3:**
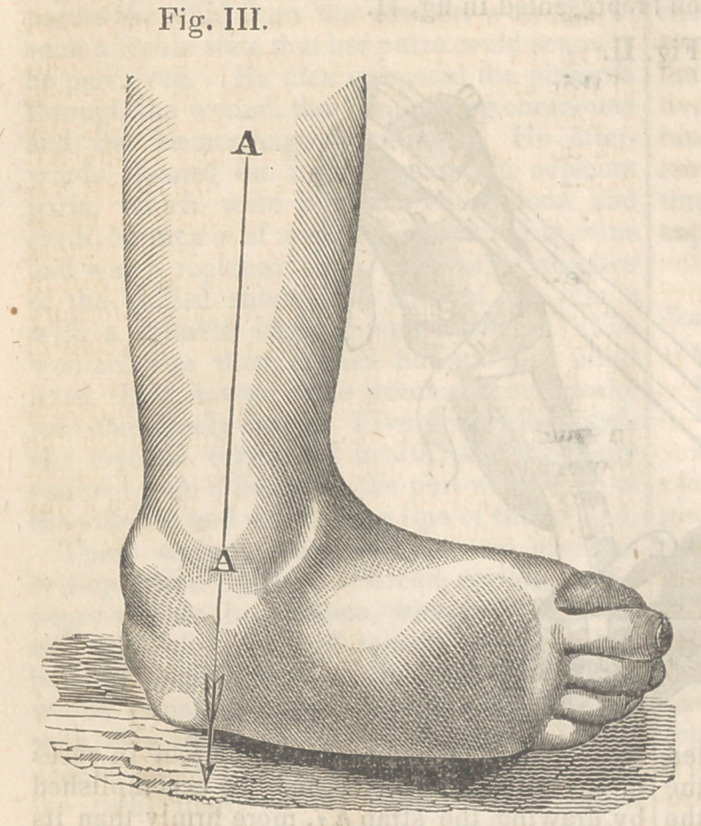


**Fig. IV. f4:**
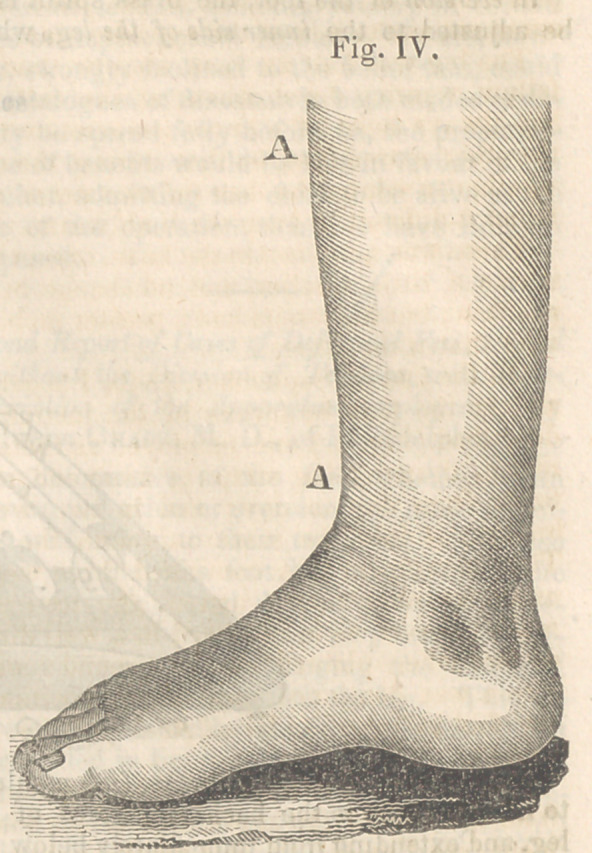


**Fig. V. f5:**
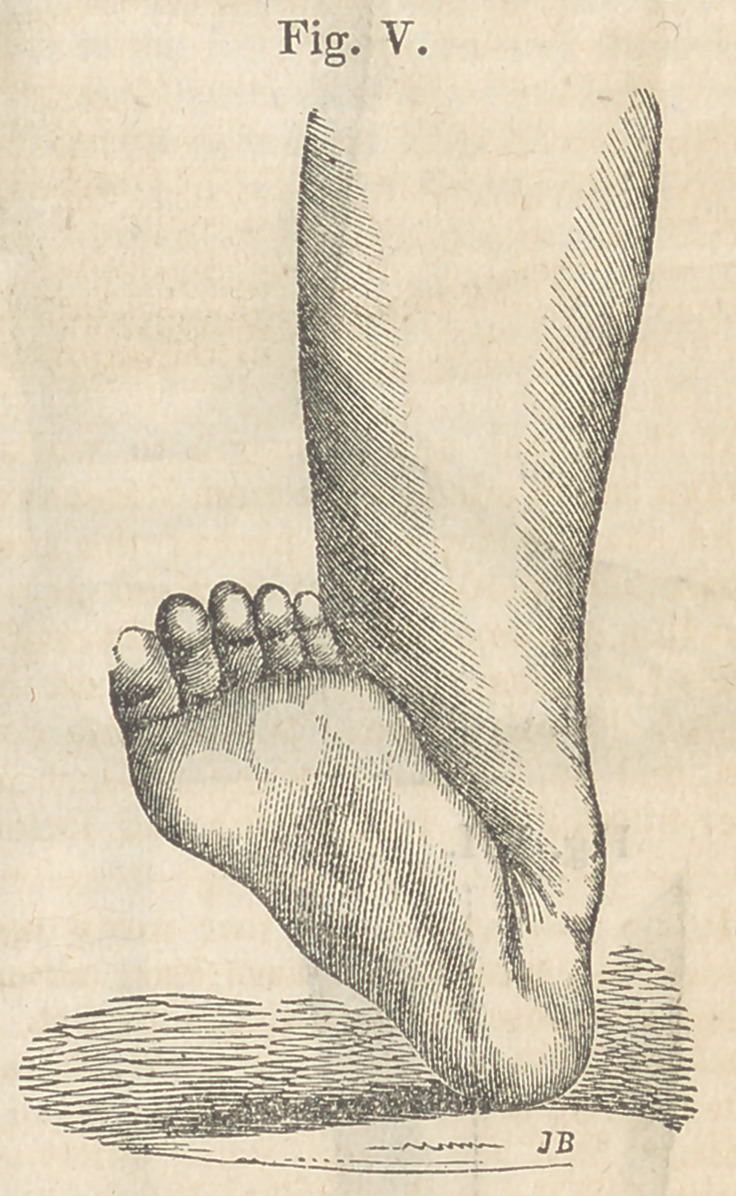


**Fig. VI. f6:**
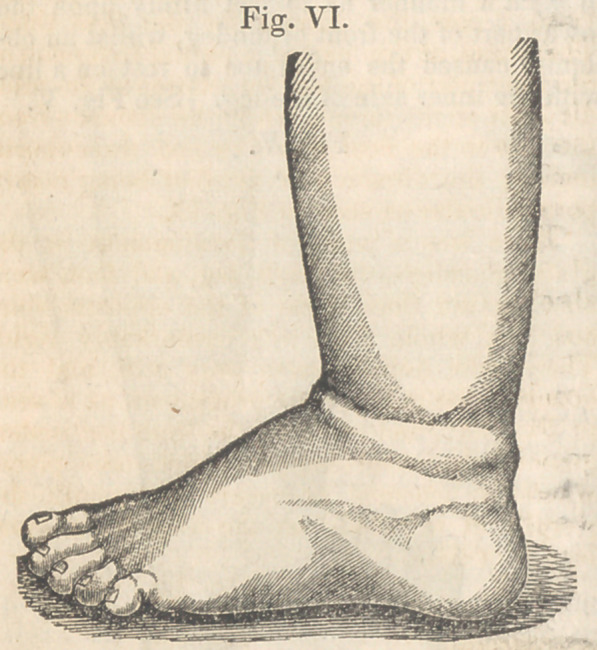


**Fig. VII. f7:**
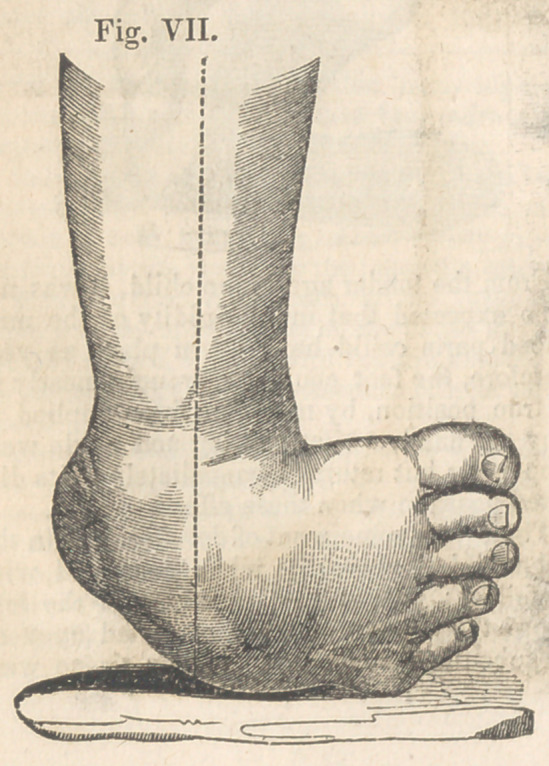


**Fig. VIII. f8:**
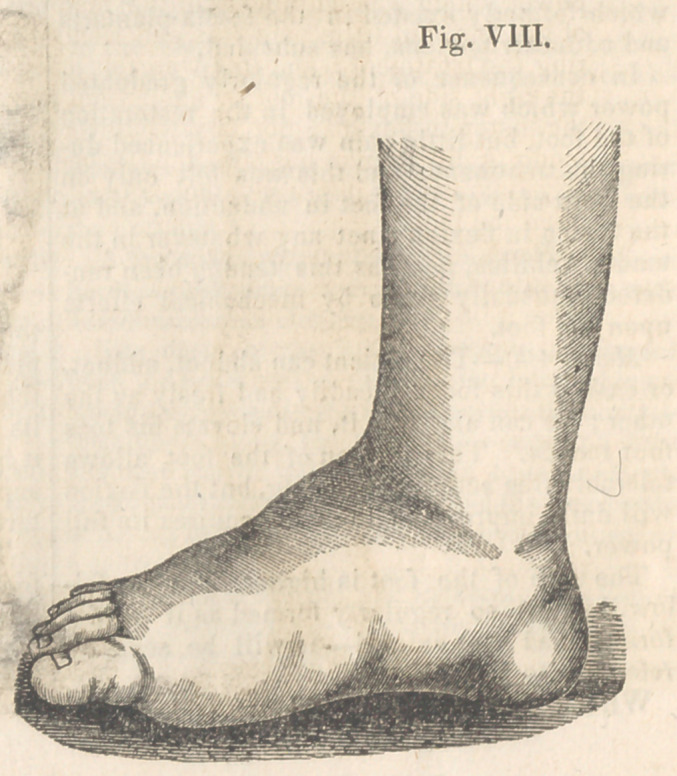


**Fig. IX. f9:**
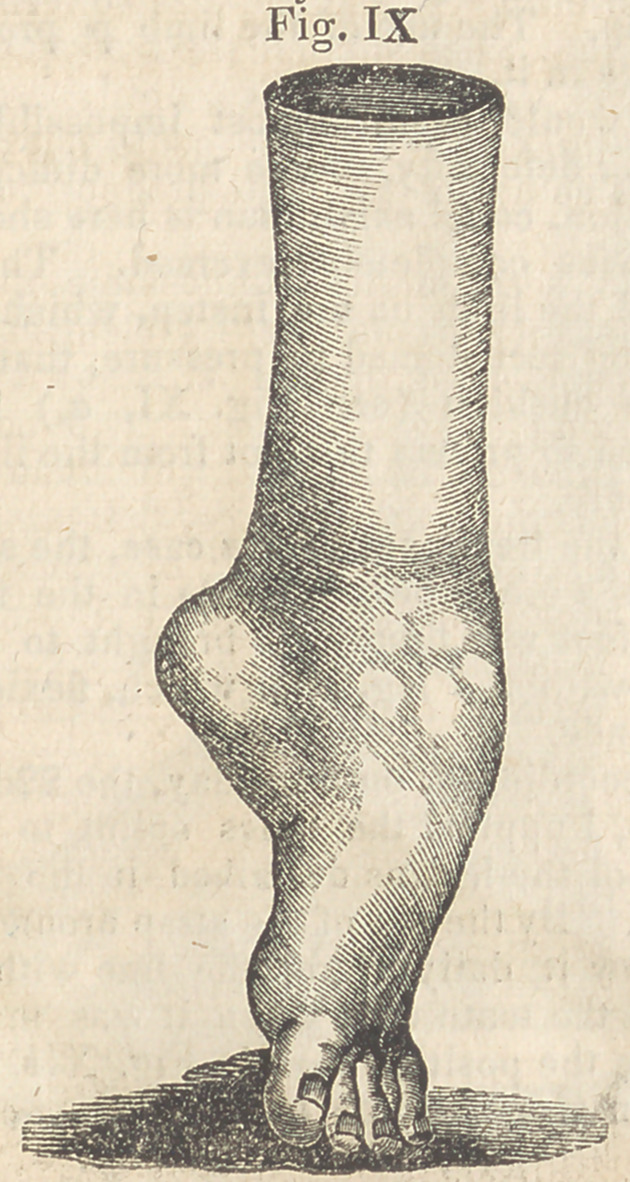


**Fig. X. f10:**
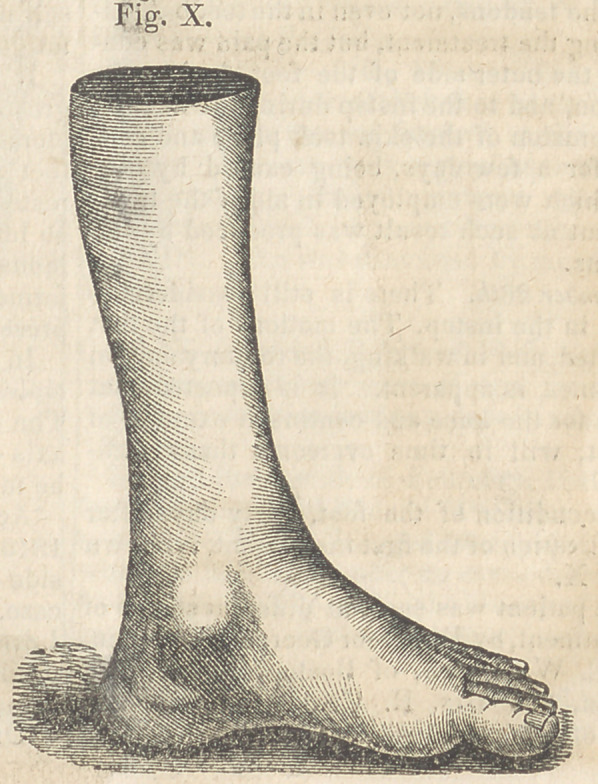


**Fig. XI. f11:**
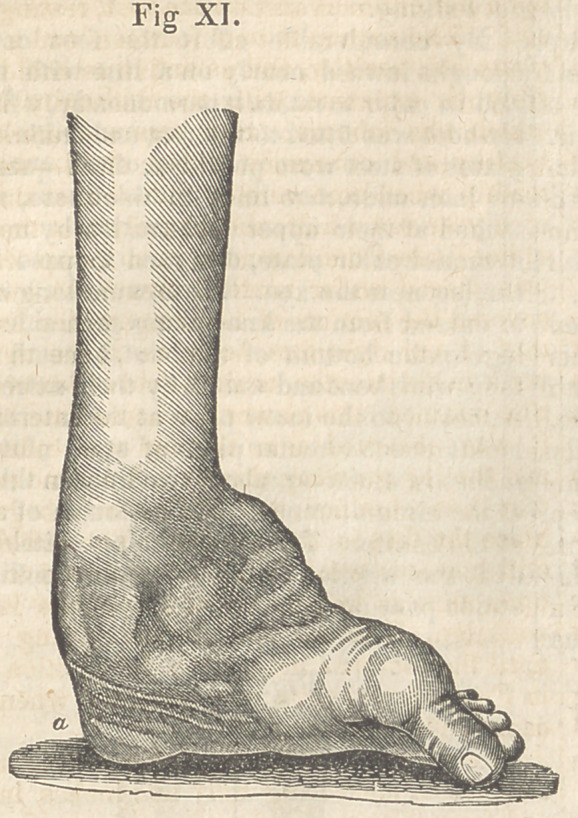


**Fig. XII. f12:**
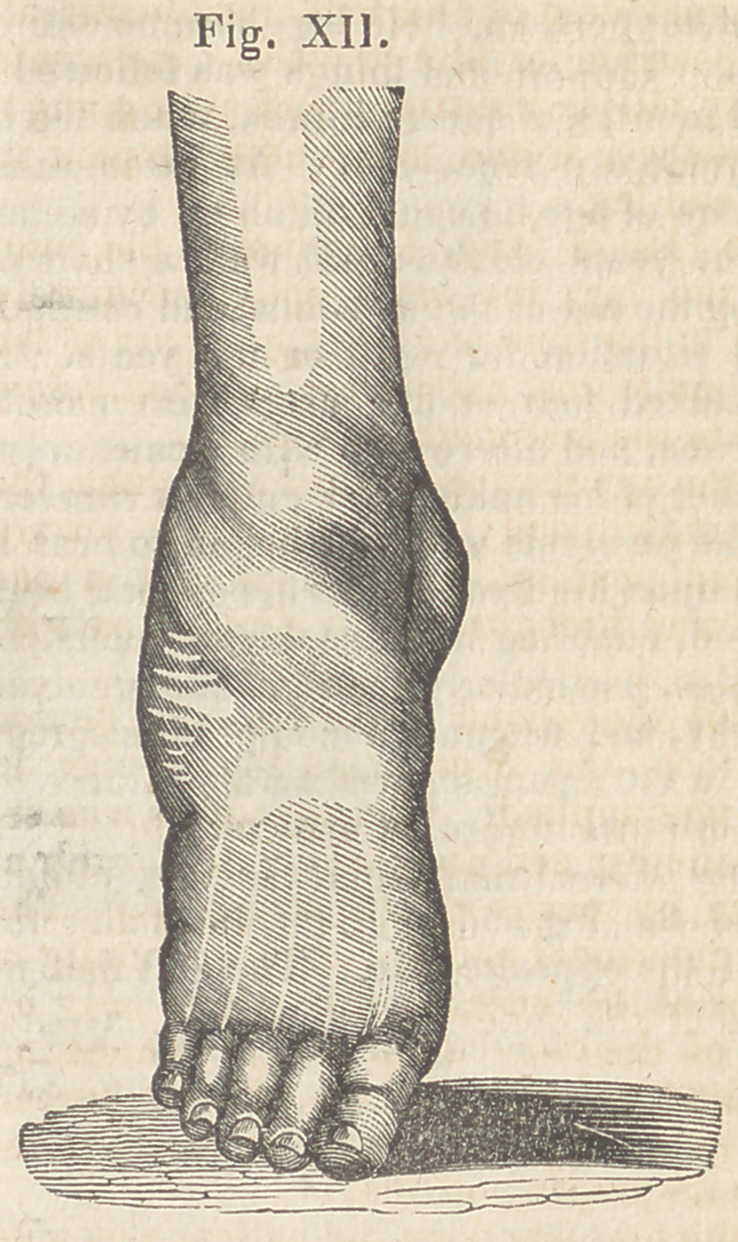


**Fig. XIII. f13:**
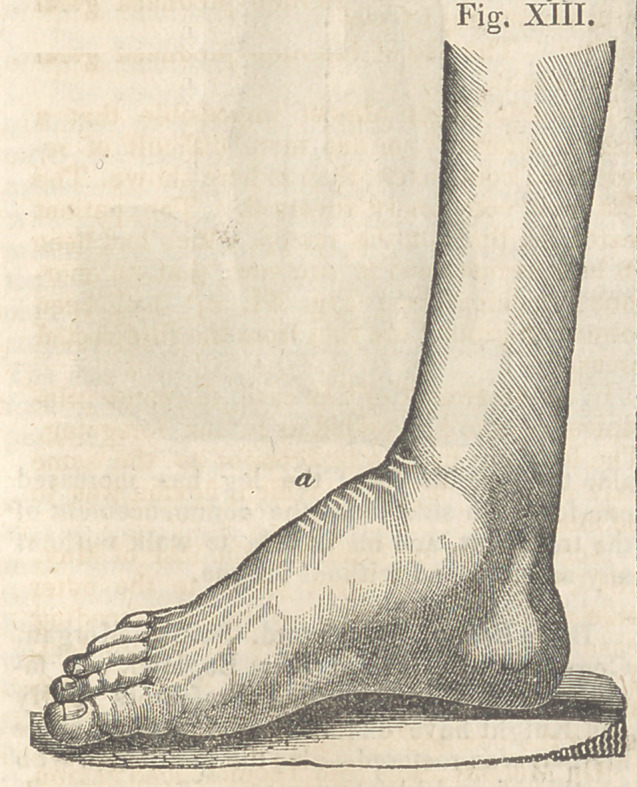


**Fig. XIV. f14:**
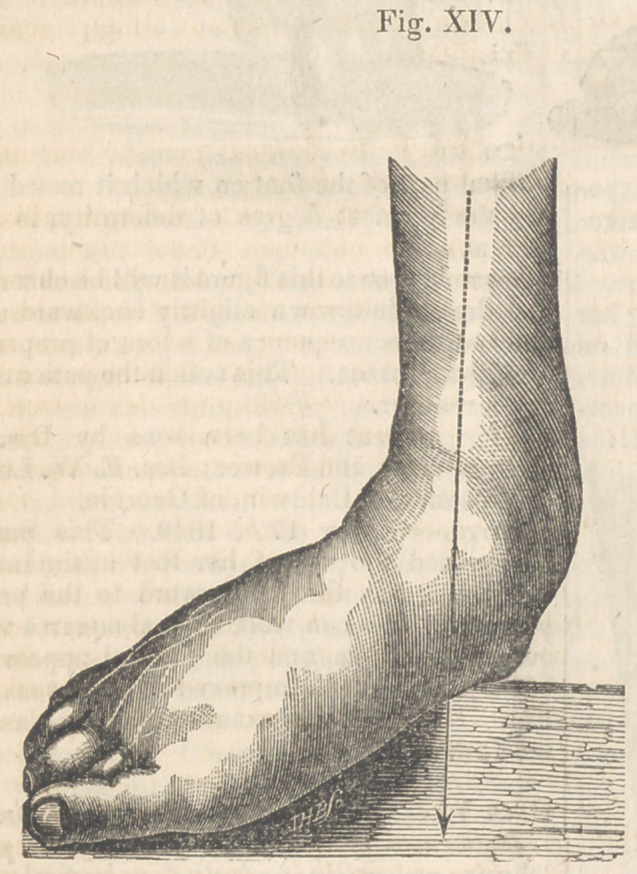


**Fig. XV. f15:**
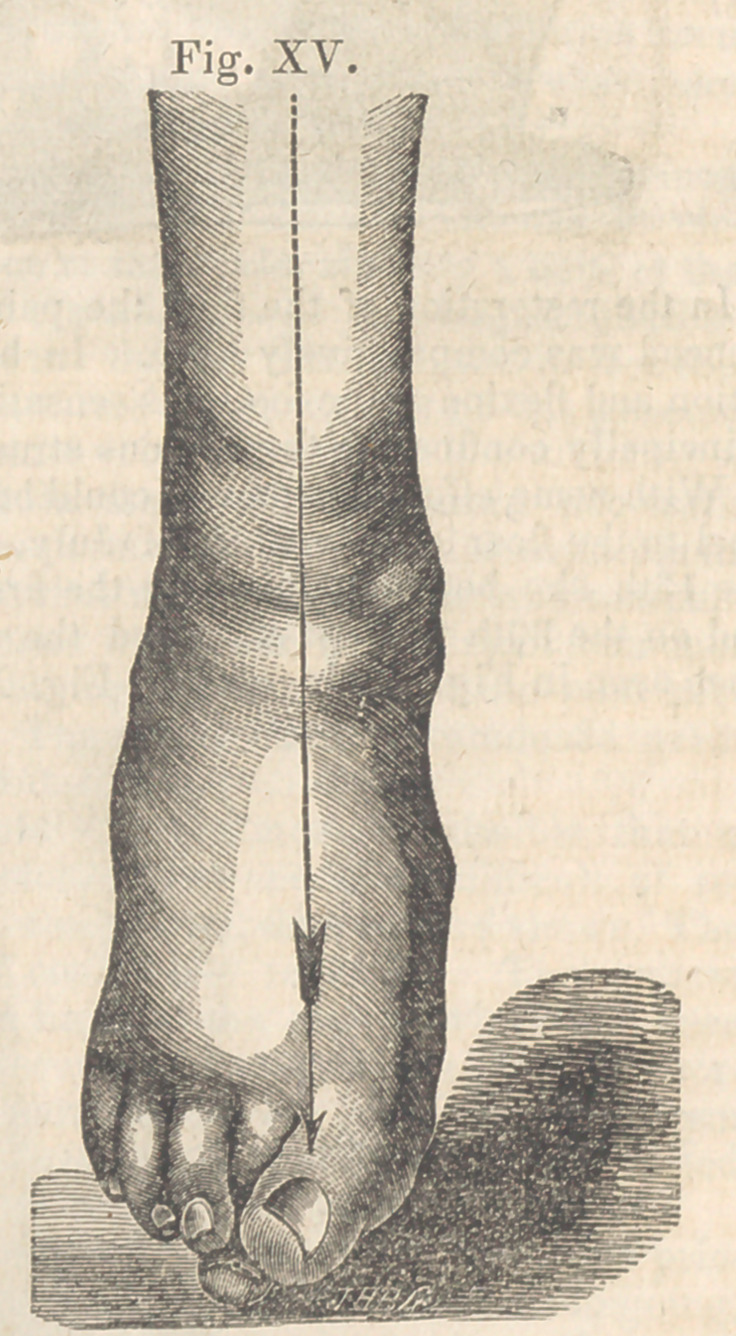


**Fig. XVI. f16:**